# Resistance to aztreonam-avibactam due to CTX-M-15 in the presence of penicillin-binding protein 3 with extra amino acids in *Escherichia coli*

**DOI:** 10.3389/fmicb.2022.1047109

**Published:** 2022-11-04

**Authors:** Ke Ma, Zhiyong Zong

**Affiliations:** ^1^Center of Infectious Diseases, West China Hospital, Sichuan University, Chengdu, China; ^2^Center for Pathogen Research, West China Hospital, Sichuan University, Chengdu, China; ^3^Division of Infectious Diseases, State Key Laboratory of Biotherapy, Chengdu, China

**Keywords:** resistance, avibactam, aztreonam, CTX-M, *Escherichia coli*

## Abstract

Aztreonam-avibactam is a promising combination to treat carbapenem-resistant Enterobacterales including coverage for metallo-β-lactamases. *Escherichia coli* strains resistant to aztreonam-avibactam have emerged but resistance mechanisms remain to be elucidated. We performed a study to investigate the mechanism for aztreonam-avibactam in a carbapenem-resistant *Escherichia coli* clinical strain. This strain was resistant to aztreonam-avibactam (aztreonam MIC, 16 mg/L in the presence of 4 mg/L avibactam). Whole genome sequencing revealed that the strain carried metallo-β-lactamase gene *bla*_NDM-4_ and the extended-spectrum β-lactamase (ESBL) gene *bla*_CTX-M-15_ and had a YRIK four amino acid insertion in penicillin-binding protein 3 (PBP3). *bla*_CTX-M-15_ was cloned into pET-28a(+), followed by the transformation, with the gene, of *E. coli* strain 035125∆pCMY42 possessing the YRIK insertion in PBP3 and strain BL21 with the wildtype PBP3. *bla*_CTX-M-14_, another common ESBL gene, and *bla*_CTX-M-199_, a hybrid of *bla*_CTX-M-14_ and *bla*_CTX-M-15_ were also individually cloned into both *E. coli* strains for comparison. Aztreonam-avibactam resistance was only observed in the *E. coli* strains with the YRIK insertion in PBP3 that produced CTX-M-15 or its hybrid enzyme CTX-M-199. Checkerboard titration assays were performed to determine the synergistic effects between aztreonam-avibactam and ceftazidime or meropenem. Doubling avibactam concentration *in vitro* reversed aztreonam-avibactam resistance, while the combination of aztreonam-avibactam and ceftazidime or meropenem did not. In conclusion, CTX-M enzymes with activity against aztreonam, (e.g., CTX-M-15 and CTX-M-199), can confer resistance in the combination of PBP3 with YRIK insertions in metallo-β-lactamase-producing carbapenem-resistant *E. coli*. Doubling the concentration of avibactam may overcome such resistance.

## Introduction

Production of metallo-β-lactamases (MBLs), in particular NDM, is a major mechanism mediating resistance to carbapenems in *Escherichia coli* (Zhang R. et al., 2017; Wang et al., 2018), a member of the order Enterobacterales. MBLs can also hydrolyze cephalosporins and penicillins but not aztreonam (ATM), a monobactam (Yong et al., 2009; Nordmann et al., 2011). However, MBL-producing carbapenem-resistant Enterobacterales often produces extended-spectrum β-lactamases (ESBLs; e.g., CTX-M enzymes) and/or AmpC cephalosporinases such as CMY enzymes, both of which are serine-based enzymes, and therefore confer resistance to ATM (Wu et al., 2019). Currently, no inhibitors of MBLs have been approved for clinical use. Avibactam (AVI), a non-β-lactam β-lactamase inhibitor, is able to inhibit serine-based β-lactamases but not MBLs (Livermore et al., 2011). The combination of aztreonam-avibactam (ATM-AVI) has activity against isolates producing either serine-based β-lactamases or MBLs or both and has been recommended to treat MBL-producing carbapenem-resistant Enterobacterales (Tamma et al., 2021; Paul et al., 2022).

However, ATM-AVI resistance has also emerged in *E. coli* (Alm et al., 2015; Russ et al., 2020) but the mechanism for resistance remains poorly understood. Several studies have demonstrated that the insertion of four amino acids (YRIN, YRIK, or YRIP) in penicillin-binding protein 3 (PBP3) leads to reduced susceptibility to ATM-AVI but does not reach the breakpoint of clinical resistance (ATM MIC ≥16 mg/L in the presence of AVI; Alm et al., 2015; Zhang Y. et al., 2017; Ma et al., 2020; Mendes et al., 2021; Ma et al., 2022). The insertion of four amino acids in PBP3 is due to a duplication of a 12-nucleotide sequence, TATCGAATTAAC for YRIN, TATCGAATTAAA for YRIK, or TATCGCATTCCT for YRIP, in the encoding gene *ftsI* (Alm et al., 2015; Ma et al., 2020; Wang et al., 2022) and the three types of insertion have the same impact on the susceptibility to ATM-AVI (Ma et al., 2020). When any of these four amino acid insertions are present in PBP3, the AmpC enzyme CMY-42 but not CMY-2 can confer resistance to ATM-AVI (Ma et al., 2020; Sadek et al., 2020). CMY-42 differs from CMY-2 by an amino acid substitution (S211V, Ambler position; Hentschke et al., 2011), which renders 8-fold higher MIC against ATM (Ma et al., 2020). The discrepancy between CMY-42 and CMY-2 in ATM-AVI resistance suggests that other β-lactamases with significant activity against ATM could involve in such resistance. CTX-M-14 and CTX-M-15 are the two major types of ESBLs in *E. coli* (Bevan et al., 2017). In this study, we report that CTX-M-15 but not CTX-M-14 can confer resistance to ATM-AVI in the presence of PBP3 with a YRIK insertion in *E. coli*, representing a new resistance mechanism of clinical significance.

## Materials and methods

### Strains and *in vitro* susceptibility testing

We have previously reported three ST405 carbapenem-resistant *E. coli* clinical isolates, WCHEC96200, WCHEC1837, and WCHEC99540, of the same clone (Zhang et al., 2018). As the three isolates carry *bla*_NDM-4_, we tested their *in vitro* susceptibilities to ATM and ATM-AVI using broth microdilution according to the Clinical and Laboratory Standards Institute (CLSI; CLSI, 2022) and the breakpoints of ATM were applied for ATM-AVI as there were no available criteria for ATM-AVI. *In vitro* susceptibility testing throughout the study was performed in triplicate.

### Cloning experiment

The −10, and −35 boxes within the promotor of *bla*_CTX-M-14_, *bla*_CTX-M-15_ and *bla*_CTX-M-199_ were predicted using BPROM.^1^ Each*bla*_CTX-M_ gene and corresponding promoter sequence were amplified using PrimeSTAR Max DNA Polymerase (Takara; Dalian, China) and primers which contained restriction sites (sequences underlined) for cloning into. pET-28a(+; Miaolingbio; Wuhan, China). *bla*_CTX-M-14_ and its promoter sequence were amplified from a local *E. coli* isolate with primers NdeI-ctx14-up (5’-AATCATATGCATCAGCAAAAGGGGATGAT-3′; the restriction site is underlined in all primer sequences) and EcoRI-ctx14-dw (5’-AAAGAATTCCTGCGTTGTCGGGAAGATAC-3′). *bla*_CTX-M-15_ and its promoter sequence were amplified from isolate WCHEC96200 (Zhang et al., 2018) with primers NdeI-ctx15-up (5′-AATCATATGCGGTGGGTCATCTCTTGCTA-3′) and EcoRI-ctx15-dw (5′- AAAGAATTCCCAGGAACCACGGAGCTTAT-3′). *bla*_CTX-M-199_ and its promoter sequence were amplified with primers NheI-ctx199-up (5′-AAAGCTAGCTGAAAAGCGTGGTAATGCTG-3′) and BamHI-ctx199-dw (5′-AAAGGATCCGAGCTTATGGCCTGGTATGC-3′) from strain WCHEC025970, which we have previously reported (GenBank accession no. CP036178).

Amplicons were purified and digested with the appropriate restriction enzyme combination (*Nde*I and *EcoR*I or *Nhe*I and *BamH*I; Takara) and were individually ligated into similarly digested fragments of the vector pET-28a(+; Miaolingbio; Wuhan, China)using T4 ligase (Takara). The resultant plasmid constructs pET-CTXM14, pET-CTXM15, and pET-CTXM199, were individually transformed into *E. coli* strain BL21 (with wild-type PBP3) and strain 035125△pCMY42 (possessing a YRIK insertion in PBP3), a ST410 *E. coli* strain cured of the plasmid carrying *bla*_CMY-42_ (Ma et al., 2020), respectively, using the chemical method. Potential transformants were screened on LB agar plates containing 50 mg/L kanamycin. The presence of *bla*_CTX-M-14_, *bla*_CTX-M-15_, and *bla*_CTX-M-199_ in the corresponding transformants, BL21::CTX-M-14, BL21::CTX-M-15, BL21::CTX-M-199, 035125△pCMY42::CTX-M-14, 035125△pCMY42::CTX-M-15, and 035125△pCMY42::CTX-M-199 were verified by PCR using Primers T7 (5′-TAATACGACTCACTATAGGG-3′) and T7ter (5′-TGCTAGTTATTGCTCAGCGG-3′) and subsequent Sanger sequencing. Minimum inhibitory concentrations (MICs) of ATM and ATM-AVI against the transformants were determined by broth microdilution (CLSI, 2022).

### Alignment of CTX-M-14, CTX-M-15, and CTX-M-199

Amino acid sequences of the three CTX-M enzymes were aligned using Clustal Omega^2^ with default settings.

### Checkerboard titration assays

Checkerboard titration assays were performed as described previously (Eucast, 2000) for the *bla*_NDM-4_-carrying isolate WCHEC96200 (Zhang et al., 2018) to determine the synergistic effects between ATM-AVI and ceftazidime (CAZ) or meropenem (MEM) in 96-well microtiter plates. Antimicrobial agents were diluted by Cation-adjusted Mueller Hinton Broth (Haibo; Qingdao, China) to obtain eight two-fold serial concentration gradients from the concentrations equal to the MICs of each agent or 1,024 mg/L if MIC was >1,024 mg/L. The fractional inhibitory concentration index (FICI) was calculated by the following: (MIC of ATM-AVI in combination with [CAZ or MEM])/(MIC of ATM-AVI) + (MIC of [CAZ or MEM] in combination with ATM-AVI)/(MIC of CAZ or MEM; Eucast, 2000). The calculated FICI was defined as synergism (≤ 0.5), indifference (> 0.5 to ≤4.0) and antagonism (> 4.0), respectively as described previously (Eucast, 2000).

### *In vitro* susceptibility testing with doubling concentrations of AVI

MICs of ATM against strain WCHEC96200 were also determined in the presence of 8 mg/L AVI (rather than 4 mg/L in CLSI recommendations) using broth microdilution (CLSI, 2022).

## Results

The three ST405 carbapenem-resistant *E. coli* clinical isolates that carried *bla*_NDM-4_ (Zhang et al., 2018) were resistant to ATM (MIC >1,024 mg/L) and ATM-AVI (MIC 16/4 mg/L). Like other NDM variants, NDM-4 has no significant activity against ATM (Nordmann et al., 2012) and therefore its presence cannot explain the resistance to ATM and ATM-AVI. Draft genome sequences of the three isolates (GenBank accession no. NGUU00000000, NGUV00000000 and NGUW00000000; Zhang et al., 2018) were examined to identify potential mechanisms associated with ATM-AVI resistance. All three isolates had a four amino acid (YRIK) insertion in PBP3 and carried *bla*_CTX-M-15_, which is able to hydrolyze ATM (Zhang et al., 2018), but can be inhibited by AVI (Livermore et al., 2011). Since *bla*_CTX-M-15_ alone cannot explain the ATM-AVI resistant phenotype in the isolates, we hypothesized that the presence of PBP3 with a YRIK insertion is playing a contributing role. As such, cloning experiments were performed to examine this hypothesis.

*bla*_CTX-M-15_ was individually cloned in *E. coli* strain BL21 (with wild-type PBP3) and strain 035125△pCMY42 (with a YRIK insertion in PBP3), to examine whether *bla*_CTX-M-15_ is able to mediate ATM-AVI resistance in either PBP3 background. BL21::CTX-M-15 was resistant to ATM but not to ATM-AVI, while 035125△pCMY42::CTX-M-15 was resistant to both (Table 1). We noted that The European Committee on Antimicrobial Susceptibility Testing (EUCAST)^3^ uses different breakpoints for aztreonam (MIC, ≤1 mg/L for ‘susceptible’ and > 4 mg/L for ‘resistant’) from CLSI (≤4 mg/L and ≥ 16 mg/L, respectively). Even using EUCAST breakpoints, 035125△pCMY42::CTX-M-14 was still not resistant to aztreonam-avibactam (4 mg/L). The above findings suggest that the combination of PBP3 with a YRIK amino acid insertion and *bla*_CTX-M-15_ can mediate resistance to ATM-AVI, while *bla*_CTX-M-15_ alone cannot.

**Table 1:** MICs (mg/L) of ATM and ATM-AVI against strains used in this study.

Strain	ATM	ATM-AVI
WCHEC96200	**>1,024**	**16**
BL21	0.015	0.015
BL21:pET-28	0.015	0.015
BL21::CTX-M-14	**64**	0.015
BL21::CTX-M-15	**128**	0.03
BL21::CTX-M-199	**128**	0.03
035125△pCMY42	2	1
035125△pCMY42::pET-28	2	1
035125△pCMY42::CTX-M-14	**64**	4
035125△pCMY42::CTX-M-15	**128**	**16**
035125△pCMY42::CTX-M-199	**128**	**16**

MICs that have reached or exceeded the resistant breakpoints are highlighted in bold.

*bla*_CTX-M-14_ is another major type of *bla*_CTX-M_ in the world (Bevan et al., 2017) and therefore we sought to examine whether *bla*_CTX-M-14_ could mediate resistance to ATM-AVI in the presence of PBP3 with a YRIK insertion like *bla*_CTX-M-15_. As expected, the obtained transformant BL21::CTX-M-14 was resistant to ATM (MIC, 64 mg/L) but not to ATM-AVI (MIC, 0.015/4 mg/L). However, 035125△pCMY42::CTX-M-14 was also susceptible to ATM-AVI (MIC, 4/4 mg/L), although the susceptibility was reduced by four-fold (from 1/4 to 4/4 mg/L; Table 1). This suggests that the combination of PBP3 amino acid insertion and *bla*_CTX-M-14_ is able to confer reduced susceptibility to ATM-AVI, but the level does not reach the breakpoint to define clinical resistance. In other words, the combination of PBP3 amino acid insertion and *bla*_CTX-M-14_ is still inadequate to confer resistance to ATM-AVI.

**Figure 1:**
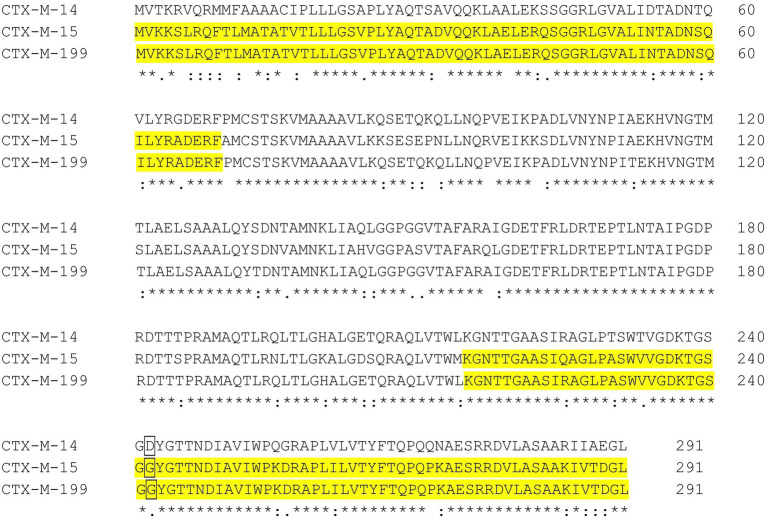
Alignment of CTX-M-14, CTX-M-15 and CTX-M-199. CTX-M-199 is a hybrid comprising the N- and C-termini of CTX-M-15 (highlighted in yellow) and the middle part of CTX-M-14. D in CTX-M-14 and G in CTX-M-15 and CTX-M-199 at the Amber position 240 are shown in framed letters. Position numbers shown in the figure are according to the start codon.

Previously, we have found *bla*_CTX-M-199_ in an *E. coli* clinical strain (WCHEC025970, GenBank accession no. CP036178). CTX-M-199 is a hybrid β-lactamase comprising the N and C termini of CTX-M-15 and the middle part of CTX-M-14 (Cai et al., 2017; Figure 1). MICs of ATM and ATM-AVI against transformants containing *bla*_CTX-M-199_ were identical to those containing *bla*_CTX-M-15_ (Table 1). Of note, the promoter sequences of *bla*_CTX-M-14_, *bla*_CTX-M-15_, and *bla*_CTX-M-199_ are identical (Supplementary Figure). The identical ATM and ATM-AVI MICs suggest that the key amino acid residues involved with resistance to ATM are likely located in the N and C termini of CTX-M enzymes.

To explore potential approaches to overcome ATM-AVI conferred by PBP3 amino acid insertion and CTX-M-15 in carbapenem-resistant *E. coli* strains, we attempted two approaches. First, we performed checkerboard titration assays that assessed the activity of ATM-AVI in combination with CAZ [as synergy among these has been reported before (Wenzler et al., 2017)] or MEM [as it is not hydrolyzed by CTX-M-15 and susceptibility is not impaired by PBP3 amino acid insertion (Alm et al., 2015)] on the *bla*_NDM-4_-carrying strain WCHEC96200. No synergistic effects between ATM-AVI and CAZ or MEM with >1 or 1.5 fractional inhibitory concentration index (FICI; Table 2). Second, we increased the concentration of AVI from 4 to 8 mg/L and found that in the presence of 8 mg/L AVI, the MIC of ATM for WCHEC96200 reduced from 16 to 4 mg/L, which is in the susceptible range. This suggests that doubling the concentration of AVI was able to overcome ATM-AVI resistance seen in *E. coli* producing CTX-M-15 and with the YRIK insertion in PBP3.

**Table 2:** Checkerboard results (mg/L) of ATM-AVI in combination with MEM or CAZ.

ATM-AVI	MEM									CAZ								
	1,024	512	256	128	64	32	16	8	0	1,024	512	256	128	64	32	16	8	0
32/4	−	−	−	−	−	−	−	−	−	−	−	−	−	−	−	−	−	−
16/4	−	−	−	−	−	−	−	−	−	−	−	−	−	−	−	−	−	−
8/4	−	−	+	+	+	+	+	+	+	+	+	+	+	+	+	+	+	+
4/4	−	−	+	+	+	+	+	+	+	+	+	+	+	+	+	+	+	+
2/4	−	−	+	+	+	+	+	+	+	+	+	+	+	+	+	+	+	+
1/4	−	−	+	+	+	+	+	+	+	+	+	+	+	+	+	+	+	+
0.5/4	−	−	+	+	+	+	+	+	+	+	+	+	+	+	+	+	+	+
0.25/4	−	−	+	+	+	+	+	+	+	+	+	+	+	+	+	+	+	+
0	−	+	+	+	+	+	+	+	+	+	+	+	+	+	+	+	+	+

FICI: ATM-AVI + MEM = 16/16 + 512/1024 = 1 + 0.5 = 1.5; ATM-AVI + CAZ = 1 + >1,024/>1,024 = >1. −, no growth (also highlighted in grey background); +, growth.

## Discussion

We demonstrated that in *E. coli* possessing PBP3 with a YRIK insertion that CTX-M-15 (and the hybrid CTX-M-199) could confer resistance to ATM-AVI but not CTX-M-14. Kinetic studies have found that CTX-M-15 (Poirel et al., 2002; Faheem et al., 2013) has strong hydrolytic activity to ATM and is able to confer high-level resistance to this agent, while CTX-M-14 has much weaker activity against ATM (Dutour et al., 2002; Ma et al., 2002; Ishii et al., 2007). The kinetic analysis of CTX-M-199 against ATM has not been reported (Cai et al., 2017; Chen et al., 2021) but we found that *E. coli* transformants containing *bla*_CTX-M-199_ was resistant to ATM (MIC, 128 mg/L), suggesting that CTX-M-199 is able to efficiently hydrolyze this agent. Therefore, in the presence of PBP3 with an amino acid insertion, ESBLs able to hydrolyze ATM efficiently can confer resistance to ATM-AVI. Previous studies have found that Ambler position 240 of class A β-lactamases was critical for hydrolyzing CAZ and ATM (Cantu et al., 1996; Bonnet et al., 2000). In this position, CTX-M-14 has a D, while CTX-M-15 and CTX-M-199 has a G (Figure 1) and it has been well documented that the D240G amino acid substitution of CTX-M enzymes enhances hydrolysis of CAZ (Bonnet et al., 2003; Cartelle et al., 2004; Celenza et al., 2008; Novais et al., 2008). ATM and CAZ share an identical bulky oxyimino R1 side-chain connected the β-lactam ring (Mitchell et al., 2015). The D240G substitution has also been found to enhance activity against ATM by increasing the affinity (Bonnet et al., 2001; Poirel et al., 2002; Cartelle et al., 2004). In addition, CTX-M-15 appears to have a higher *k_cat_* (1.5 S^−1^; Poirel et al., 2002) compared to CTX-M-14 (*k_cat_*, <0.01 S^−1^; Ma et al., 2002). This suggests that CTX-M-15 is likely to hydrolyze ATM more rapidly than CTX-M-14, contributing to the stronger activity of the former enzyme against ATM.

It is known that the inhibition of β-lactamases by AVI is reversible by recyclization (Ehmann et al., 2012; Lahiri et al., 2014) and usually does not result in hydrolysis (Lahiri et al., 2014). This suggests that some free forms of CTX-M enzymes are likely to exist in the presence of AVI and are therefore available to attack ATM. As described previously, the four-amino-acid insertion is located in the tight turn between the β2b–β2c sheets adjacent to the β-lactam binding pocket pf PBP3, resulting in reduced affinity to PBP3-binding β-lactams such as ATM (Alm et al., 2015). The reduced affinity of PBP3 increases the amount of ATM exposed to CTX-M enzymes, which may need higher concentrations of AVI to protect the increased amount of ATM from the enzymatic hydrolysis. This could explain that doubling the concentration of AVI is able to reverse ATM-AVI resistance conferred by CTX-M-15 and CTX-M-199.

We are aware of limitations of our study. First, we used strain 035125△pCMY42 for studying the impact of CTX-M in the presence of a four-amino-acid insertion in PBP3 and strain BL21 for studying that of CTX-M in the absence of such an insertion. The two strains have a different genetic background, which introduces confounding factors for the cloning experiments. Nonetheless, we compared 035125△pCMY42 with and without *bla*_CTX-M_ and then compared BL21 with and without *bla*_CTX-M_. The two pairs of comparisons support the presence of joint effects of CTX-M enzymes and the PBP3 alteration on the susceptibility to ATM-AVI. Second, we did not perform experiments to investigate the interaction among CTX-M enzymes, AVI, and PBP3 with or without the four-amino-acid insertion. Therefore, the exact mechanisms that CTX-M-15 and CTX-M-199 confer resistance to ATM-AVI while CTX-M-14 leads to reduced susceptibility in *E. coli* possessing a four-amino-acid insertion in PBP3 are still not clear. Nonetheless, the observation that production of CTX-M-15, a very common ESBL, could confer resistance to ATM-AVI in *E. coli* with PBP3 insertion, which is increasingly reported (Mendes et al., 2021; Wang et al., 2022) is clinically relevant. Third, we did not perform *in vivo* studies to verify that the ATM-AVI resistance could be reversed by increasing the concentration of AVI. The clinical implications for this *in vitro* observation remain to be investigated.

In conclusion, CTX-M enzymes with significant activity against ATM (e.g., CTX-M-15 and CTX-M-199) can confer resistance in MBL-producing carbapenem-resistant *E. coli* possessing the four-amino-acid insertion PBP3, further compromising therapeutic options. Doubling the concentration of AVI may overcome such resistance. Further studies of ATM-AVI resistance mechanisms in MBL-producing Enterobacterales are warranted to inform clinical practice.

## Data availability statement

The datasets presented in this study can be found in online repositories. The names of the repository/repositories and accession number(s) can be found at: https://www.ncbi.nlm.nih.gov/, NGUU00000000; https://www.ncbi.nlm.nih.gov/, NGUV00000000; https://www.ncbi.nlm.nih.gov/, NGUW00000000.

## Author contributions

ZZ conceived and designed the study. KM performed the experiments. KM and ZZ analyzed the data and wrote the manuscript. All authors contributed to the article and approved the submitted version.

## Funding

The relevant works of the authors were supported by the National Natural Science Foundation of China (82172309 and 81861138055) and by grants from the West China Hospital of Sichuan University (1.3.5 project for disciplines of excellence, grant number ZYYC08006 and ZYGD22001).

## Conflict of interest

The authors declare that the research was conducted in the absence of any commercial or financial relationships that could be construed as a potential conflict of interest.

## Publisher’s note

All claims expressed in this article are solely those of the authors and do not necessarily represent those of their affiliated organizations, or those of the publisher, the editors and the reviewers. Any product that may be evaluated in this article, or claim that may be made by its manufacturer, is not guaranteed or endorsed by the publisher.

## Footnotes

^1^ http://www.softberry.com/berry.phtml?topic=bprom&group=programs&subgroup=gfindb

^2^ https://www.ebi.ac.uk/Tools/msa/clustalo/

^3^ https://www.eucast.org
