# Supplementary material for: Resistance to aztreonam-avibactam due to CTX-M-15 in the presence of penicillin-binding protein 3 with extra amino acids in Escherichia coli
Source: Front Microbiol. 2022 Nov 4;13:1047109. doi: 10.3389/fmicb.2022.1047109 (PMC9674307; doi:10.3389/fmicb.2022.1047109)
Supplement: Supplementary file 1 [file Presentation_1.PDF]

CTX-M-14	AACTTGCAGAGTTTATTAAGTATCATTGCAGCAAAGATGAAATCAATGATTTATCAAAAA
CTX-M-15	AACTTGCAGAGTTTATTAAGTATCATTGCAGCAAAGATGAAATCAATGATTTATCAAAAA
CTX-M-199	AACTTGCAGAGTTTATTAAGTATCATTGCAGCAAAGATGAAATCAATGATTTATCAAAAA
CTX-M-14	TGATTGAAAGGTGGTTGTAAATAATGTTACAATGTGTGAGAAGCAGTCTAAATTCTTCGT
CTX-M-15	TGATTGAAAGGTGGTTGTAAATAATGTTACAATGTGTGAGAAGCAGTCTAAATTCTTCGT
CTX-M-199	TGATTGAAAGGTGGTTGTAAATAATGTTACAATGTGTGAGAAGCAGTCTAAATTCTTCGT
CTX-M-14	GAAATAGTGATTTTTGAAGCTAATAAAAAA--CACGTGGAATTTAGGGAATACTGATGTA
CTX-M-15	GAAATAGTGATTTTTGAAGCTAATAAAAAACACACGTGGAATTTAGGGACTATTCATGTT
CTX-M-199	GAAATAGTGATTTTTGAAGCTAATAAAAAACACACGTGGAATTTAGGT---ATTCATGTT
CTX-M-14	AC-------ACGGATTGACC-GTATTGGGAGTTTGAG**ATGGTGACAAAGAGAGTGCAACG**
CTX-M-15	GTTGTTATTTCGTATCTTCCAGAATAAGGAAT--CCC**ATGGTTAAAAAATCACTGCGCCA**
CTX-M-199	GTTGTTATTTCGTCTCTTTCAGAATAAGGAAT--CCC**ATGGTTAAAAAATCACTGCGCCA**

Figure S1. The promoter sequences of *bla*$_{CTX\text{-}M\text{-}14}$, *bla*$_{CTX\text{-}M\text{-}15}$, and *bla*$_{CTX\text{-}M\text{-}199}$. The -10 and -35 sequences are boxed, and the sequences of *bla*$_{CTX\text{-}M}$ genes are in bold.
